# Correlation between maternal eating disorder and early infant feeding regulation: a cross -sectional study

**DOI:** 10.1186/s12884-021-04317-z

**Published:** 2021-12-20

**Authors:** Rachel F. Rodgers, Morgan Hines, Alaina Martens, Emily Zimmerman

**Affiliations:** 1 APPEAR, Department of Applied Educational Psychology, Northeastern University, 360 Huntington Avenue, Boston, MA 02115 USA; 2 Department of Psychiatric Emergency & Acute Care, Lapeyronie Hospital, CHRU Montpellier, 291 Av. du Doyen Gaston Giraud, 34000 Montpellier, France; 3 Speech and Neurodevelopment Lab, Department of Communication Sciences and Disorders, Northeastern University, 360 Huntington Avenue, Boston, MA 02115 USA

**Keywords:** Postpartum, Feeding regulation, Mothers, Gender, Infant

## Abstract

**Background:**

The post-partum period is a vulnerable time for mothers in terms of eating disorder symptoms and is critical for the establishment of feeding patterns in infants. This study aimed to investigate the relationships between maternal eating disorder symptoms and objective indices of feeding regulation at 3 months, as well as perceived breastfeeding self-efficacy.

**Methods:**

A sample of *n* = 73 full-term mother-child dyads (44% female) participated in the study. Mothers self-reported eating disorder symptoms and breastfeeding self-efficacy and objective indices of infant feeding regulation were obtained in the home.

**Results:**

Findings revealed the existence of relationships between higher maternal eating disorder symptoms, and objective indices of infant feeding regulation with substantial gender differences in the patterns emerging. Among mother-daughter dyads, maternal weight and shape concerns were associated with higher infant transfer volume and rate during bottle feeding. In contrast, among mother-son dyads, higher maternal eating disorder symptoms, including weight, shape, and eating concern, were associated with lower infant transfer volume and rate as well as lower levels of proficiency while taking their bottle.

**Conclusion:**

Relationships emerged between higher maternal eating disorder symptoms and feeding regulation with substantial gender differences in these patterns. Additional research clarifying the underlying mechanisms of these associations is warranted and further efforts should be directed towards supporting mothers during the postpartum period.

## Background

An estimated 25–45% of typically developing children experience some type of feeding problem [1–3]. Such difficulties are concerning as they may persist and influence child development as well as heighten the risk for future eating concerns in children and adolescents [4]. Previous studies have documented a relation between early infant feeding difficulties and greater maternal breastfeeding difficulties, and disordered eating behaviors [1, 5, 6]. However, the mechanisms underlying these relationships are not well understood.

Importantly, conceptualizations of feeding disorders have proposed that they be framed as relational disorders, thus acknowledging that infants feeding occurs within an interaction [1, 7]. In this way, mothers with higher levels of eating concerns have also displayed lower levels of adaptation to their infants when feeding them [8], and may feel less comfortable breastfeeding. Thus, dyadic interactions during feeding may constitute an underlying mechanism for the relationship between maternal disordered eating and infant feeding difficulties. However, to date research in this area is scant. Moreover, the small amount of research that has been conducted among young infants during the first months postpartum has focused exclusively on food refusal as an outcome and has relied mainly on self-report or visual observational data for the feeding assessment [6, 8, 9]. The reliance on such methodologies limits the investigation of these mechanisms. One observational longitudinal study, however, suggested that sucking patterns in infancy predicted child pickiness between the ages of 3 and 5 years [4], highlighting the need for more work in this area.

The present study therefore sought to contribute to this area of research and investigate the relationships between maternal eating disorder symptoms and objective indices of feeding regulation at 3 months. In addition, maternal self-reported perceptions of feeding were assessed to contrast with objective indices. Given the previously documented gender-differences in feeding behaviors and practices as well as later outcomes [10, 11], we examined relationships among mother-daughter and mother-son dyads separately. Given the exploratory nature of the study, no specific directional hypotheses were formulated.

## Methods

### Aim, design, and setting

The aim of the present study was to investigate the relationships between maternal eating disorder symptoms and objective indices of feeding regulation, as well as maternal perceived breastfeeding self-efficacy in full-term infants at 3 months of age, examining these relationships between mother-daughter and mother-son dyads. We used a cross-sectional and correlational design with self-report and objective measures. The study was approved by the institutional review board (Protocol Number: 17-08-19). This study was completed in the greater Boston area with study visits occurring in participants’ homes.

### Procedures

Participants in this study were taken from a larger study that examined the relation between early sucking, oral feeding, and vocal development [12]. Parents consented for their own participation and that of their infants. Infants and their parents were recruited by word of mouth, Facebook groups, and flyer distribution. Caregivers were compensated with an Amazon gift card. Exclusion criteria for the broader study was infants born with congenital and/or chromosomal anomalies. The current study focused only on full-term infants at 3-months with primarily breastfeeding and only occasional bottle feeding (breastmilk/formula). Mothers and infants were included at 3 months post-partum as this is a period in which families typically have some established routine and feeding schedule and have not begun more complex oral movements. The study occurred in the infant’s house approximately 1 h before the infant’s scheduled feed as determined by the parent. During the study, the mother completed the eating disorder symptoms scale, the breastfeeding self-efficacy scale, and the researchers observed a bottle feed and completed the oral feeding skills (OFS) scale.

### Measures

#### Eating disorder symptoms

The Eating Disorder Examination Questionnaire (EDE-Q) 6.0 [13] focuses on the past 28 days and uses a 7-point rating scale ranging from 0 to 6, to derive scores on four subscales that have been used in non-clinical populations (Restraint, Eating Concern, Weight Concern, and Shape Concern) [14]. Higher scores indicate higher symptom levels. Here, internal consistency was alpha = .68 for restraint,^1^ .83 for weight concern, .91 for shape concern, and .73 for eating concern respectively.

#### Breastfeeding self-efficacy

Breastfeeding self-efficacy was assessed using the 14 item Breastfeeding Self-Efficacy Scale Short-Form [15]. Items begin with “I can always” and include statements such as “determine that my baby is getting enough milk.” Participants rate their agreement using a 5-point scale ranging from 1 (not at all confident) to 5 (completely confident). Scores were summed with higher scores indicating greater breastfeeding self-efficacy. Here, internal consistency was .95.

#### Feeding regulation

Feeding regulation was determined using the OFS, developed by Lau and Smith [16] as a novel approach to assess oral feeding regulation of infants. While initially created to better define oral feeding metrics with preterm infants, the scale provides a simple means of measuring certain indices of oral feeding regulation among all bottle-fed infants. The OFS was completed while observing the caregiver offer their infant the bottle. If the infant was fed breastmilk, the mother put her milk in the infant’s bottle, otherwise they used their preferred formula. No instructions were given regarding the amount of milk to provide. During the observation, a researcher denoted the following outcomes: initial volume (ml), transfer volume, (% volume taken/total volume to be taken), proficiency (% volume taken during the first 5 min/total volume prescribed), and transfer rate (ml/min).

### Statistical analyses

Given that the distribution of the EDE-Q subscales was somewhat skewed in this small, non-clinical sample, Spearman correlations were conducted. For sensitivity purposes, partial Pearson correlations controlling for infant weight were also conducted. Given the small sample size, and the exploratory nature of these data, while statistical significance (*p* < .05) is reported, effect sizes were interpreted such that coefficients between .10 and .29 represent a small association, coefficients between .30 and .49 represent a medium association, and coefficients of .50 and above represent a large association or relationship [17].

## Results

Data from a sample of *n* = 73 full-term primarily breastfeeding mother-child dyads (44% female), with an average birth weight of 3.44 kg (SD = .44), who were participating in a larger study were included. Mothers were on average 33.68 years old (SD = 3.59), with infants aged 3.02 months (SD = .30), and half of the sample (49%) were firstborns. In our cohort, 97% of parents were married, 77% were employed fulltime, and 90% had a college or graduate degree.

Mann Whitney tests revealed no significant differences between males and females across the dependent measures (see Table 1). In addition, no differences were found on variables of interest between firstborns and infants with siblings (data not shown). Among mother-daughter dyads, spearman correlations revealed that self-reported maternal weight concerns and shape concerns were moderately associated with higher transfer volume and transfer rate (see Table 2). Among mother-son dyads a different pattern emerged, with maternal weight and shape concerns moderately associated with lower transfer volume, transfer rate, and proficiency. In addition, among mother-son dyads, greater maternal eating concerns were moderately associated with lower transfer rate and proficiency. Breastfeeding self-efficacy revealed no relationships with feeding regulation while taking a bottle, with the exception of a small relationship with lower transfer rate among mother-daughter dyads. Sensitivity analyses revealed no substantial differences in the patterns of association.

**Table 1:** Descriptive statistics and gender differences for study variables

	Males *n* = 38 Median (range)	Females *n* = 32 Median (range)	*P*-value
Oral Feeding Skills			
Initial Volume	118.29 (29.57–273.56)	118.29 (29.57–177.44)	.546
Transfer Volume	73.50 (.00–100.00)	83.30 (.00–100.00)	.505
Proficiency	39.25 (.00–85.73)	35.41 (.00–100.00)	.794
Transfer Rate	8.80 (.00–22.00)	5.55 (.00–20.33)	.375
Maternal Eating Concerns			
Restraint	.20 (.00–3.20)	.10 (.00–3.40)	.472
Weight concern	1.70 (.00–4.00)	1.20 (.00–5.00)	.507
Shape concern	1.62 (.00–4.75)	1.31 (.00–4.87)	.825
Eating concern	.20 (.00–2.2)	.20 (.00–2.8)	.550
Global	1.07 (.00–3.28)	.92 (.00–3.46)	.908
Breastfeeding Self-Efficacy	59.00 (33.00–70.00)	63.00 (20.00–70.00)	1.00

Note: Mann Whitney was used for comparisons across sexes, *p < .05

**Table 2:** Bivariate associations among study variables

	Oral Feeding Skills							
	Total Volume		Transfer Volume		Proficiency		Transfer Rate	
	Girls	Boys	Girls	Boys	Girls	Boys	Girls	Boys
Maternal Eating Concerns								
Restraint	.05	.26	.13	.10	.01	.31	.17	.27
Weight concern	−.03	−.06	.34^¶^	−.38^¶^	.21	−.39*	.33^¶^	−.43*
Shape concern	−.05	−.04	.43*	−.29	.30	−.35^¶^	.43*	−.37^¶^
Eating concerns	.05	−.04	.22	−.14	.06	−.41*	.25	−.39^¶^
Breastfeeding self-efficacy	−.14	.12	−.16	.03	−.13	.17	−.25	.12

*p < .05, ^¶^*p* < .10

## Discussion

The aim of the present study was to investigate the relationships between maternal eating disorder symptoms, objective indices of feeding regulation, and maternal perceived breastfeeding self-efficacy among 3-month-old mother-daughter and mother-son dyads. Overall, findings suggest the existence of relationships between higher maternal eating disorder symptoms and feeding regulation. Moreover, substantial gender differences in these patterns emerged. Among mother-daughter dyads, as indicated by the positive correlation, maternal weight and shape concerns were associated with higher levels of infant transfer volume and rate, indicating that mothers with higher weight and shape concerns had daughters who ate more and faster, than those with lower weight and shape concerns. In contrast, among mother-son dyads, as indicated by the negative correlation, higher maternal eating disorder symptoms, including weight, shape, and eating concerns, were associated with lower levels of infant transfer volume and rate as well as lower levels of proficiency. These findings suggest that in these dyads higher maternal concerns co-occurred with sons feeding slower, less proficiently and overall eating less.

To our knowledge this is the first time the maternal eating concerns and infant feeding regulation have been examined across mother-son and mother-daughter dyads at this early time point. These findings extend the aforementioned relations between maternal disordered eating behaviors and specific infant feeding patterns including greater feeding difficulties found in previous studies [1, 6] by documenting feeding regulation in young infants. Furthermore, this study is unique in its contribution by revealing gender differences in the patterns of these relations. These gender differences are difficult to interpret in correlational data, and in the absence of other indicators such as feeding frequency. Given that no gender differences emerged in the median levels of the observed variables, it seems unlikely that infants were reacting to differences in the amount of milk provided by mothers to sons compared to daughters. It is possible that infant hunger due to differences in feeding frequency across genders might contribute to these patterns. Indeed, it has been suggested that parental concern about child weight and feeding patterns may reveal gender-biases, such that parents are more concerned about their young daughters’ weight [18] and that eating pathology has been associated with more controlling feeding practices in mothers of 3-year old daughters, but not sons [19]. However, it is notable that the pattern of associations among mother-son dyads was more consistent across indices of maternal disordered eating and feeding regulation compared to the pattern among mother-daughter dyads. Given this, it might be possible that among mothers with sons, associations between infant feeding regulation and maternal disordered eating is evident among dyads with higher levels of difficulty, characterized by maternal concerns including difficulty regulating eating behaviors, and feeding difficulties respectively. In contrast, it may be that among mother-daughter dyads, patterns are evident across lower levels of concerns and mainly reflect associations with body image concerns that could be more normative in the postpartum period. Our data suggest that this is likely a bidirectional relationship underlying these patterns that begin early in infancy and warrant future investigation across the lifespan and across feeding development.

Although the pathway is not yet clear, these relationships could be nested in the complexities of the mother-daughter relationship, as well as gendered expectations regarding eating, weight, and shape. Indeed, research has shown that mothers tend to report being more concerned about their daughters’ weight compared to that of their sons [20]. These differences likely reflect broader social differences in weight bias and overvaluation of weight and shape, with young women experiencing greater appearance pressures as compared to their male counterparts [21]. However, perhaps relatedly, research has shown that mothers perceive hunger cues differently among their breastfed female infants as compared to their male infants [22]. Therefore, the differences observed may also partly be the result of maternal feeding practices in response to their perceptions of children’s hunger cues, as well as their socialized beliefs around gender differences in those.

Interestingly, with the exception of transfer rate among female infants that revealed a small effect associated with lower maternal breastfeeding self-efficacy, no other patterns between feeding regulation and maternal perceived breastfeeding self-efficacy emerged. This is important as it suggests that while breastfeeding self-efficacy is associated with some infant feeding measures, such as infant latch and infant cues, it may not be associated with *actual* feeding proficiencies. Such a finding is consistent with the disconnect observed between maternal perceptions and feeding practices among older children [23].

This study presents a number of limitations including the cross-sectional and correlational design, the lack of maternal anthropomorphic data, and the somewhat small sample size. In addition, it was not possible to control for the time since last feeding, although parents scheduled the appointment for approximately 1 h before the predicted feeding time. Nevertheless, our study makes an important contribution to the existing literature by representing the first investigation of the relationships between maternal eating disorder symptoms and feeding regulation in very young infants.

## Conclusion

Our findings suggest that such relationships exist, although reveal interesting gender differences. Additional research confirming these patterns and investigating the underlying mechanisms is warranted. While the interpretation of these findings requires further investigation, they highlight the need for psychosocial support for mothers during the postpartum period, which is a vulnerable time for mothers who may experience heightened body image and eating concerns [24]. Furthermore, as our data suggest that maternal breastfeeding self-efficacy may be independent of their infants’ feeding behaviors, strategies to enhance breastfeeding confidence should be targeted toward the mother’s perception of the breastfeeding experience and incorporated in intervention efforts and resources.

## Data Availability

The data that support the findings of this study are available from the corresponding author upon reasonable request.

## Abbreviations

OFS scale: The Oral Feeding Skills
EDE-Q: The Eating Disorder Examination Questionnaire

## Footnotes

1 The alpha value for restraint was somewhat low, and inspection of the indices revealed that if item 2 were deleted, the alpha value would be > .70 and therefore acceptable. However, to preserve comparability of the findings here with other studies, the 5-item version of this scale was retained. Sensitivity analyses revealed that the findings were identical using the 4-item version of the Restraint scale with adequate internal reliability.
